# EZH2 inhibitors sensitize myeloma cell lines to panobinostat resulting in unique combinatorial transcriptomic changes

**DOI:** 10.18632/oncotarget.25128

**Published:** 2018-04-24

**Authors:** Taylor Harding, Jessica Swanson, Brian Van Ness

**Affiliations:** ^1^ Department of Genetics, Cell Biology and Development, University of Minnesota, Minneapolis, MN, USA

**Keywords:** myeloma, EZH2, panobinostat

## Abstract

Multiple myeloma (MM) remains a largely incurable hematologic cancer due to an inability to broadly target inevitable drug-resistant relapse. Epigenetic abnormalities are abundantly present in multiple myeloma and have increasingly demonstrated critical roles for tumor development and relapse to standard therapies. Accumulating evidence suggests that the histone methyltransferase EZH2 is aberrantly active in MM. We tested the efficacy of EZH2 specific inhibitors in a large panel of human MM cell lines (HMCLs) and found that only a subset of HMCLs demonstrate single agent sensitivity despite ubiquitous global H3K27 demethylation. Pre-treatment with EZH2 inhibitors greatly enhanced the sensitivity of HMCLs to the pan-HDAC inhibitor panobinostat in nearly all cases regardless of single agent EZH2 inhibitor sensitivity. Transcriptomic profiling revealed large-scale transcriptomic alteration by EZH2 inhibition highly enriched for cancer-related pathways. Combination treatment greatly increased the scale of gene expression change with a large portion of differentially expressed genes being unique to the combination. Transcriptomic analysis demonstrated that combination treatment further perturbed oncogenic pathways and signaling nodes consistent with an antiproliferative/pro-apoptotic state. We conclude that combined inhibition of HDAC and EZH2 inhibitors is a promising therapeutic strategy to broadly target the epigenetic landscape of aggressive MM.

## INTRODUCTION

Multiple myeloma (MM), a hematopoietic malignancy with over 30,000 new cases each year in the United States, is characterized by clonal expansion of malignant post-germinal-center B-cell-derived plasma cells within the bone marrow [1]. While current therapies including proteasome inhibitors and immunomodulatory drugs have improved disease management, MM remains largely incurable [2]. Heterogeneous patient response to therapy and the inevitable emergence of drug-resistant relapse impede long-term therapeutic efficacy. This illustrates the need for new therapeutic strategies that improve the efficacy of current compounds and more broadly target malignant plasma cells.

Epigenetic abnormalities are abundantly present in multiple myeloma (MM) and have increasingly demonstrated critical roles for tumor development and resistance to therapy [3–7]. Therapeutic strategies that target epigenetic modifiers have recently gained momentum in many cancers including recent FDA approval for the pan-HDAC inhibitor panobinostat (PAN) in MM [8–10].

Enhancer of zeste homolog 2 (EZH2), the catalytic subunit of the polycomb repressive complex 2 (PRC2), regulates the expression of thousands of genes to control developmental programs, maintain proliferative capacity and repress tumor suppressors in many forms of cancer [11–17]. EZH2’s canonical function is to repress gene expression via methylation of H3K27, however, EZH2 has recently been shown to have several additional catalytic and non-catalytic functions that regulate transcription factor complexes and non-coding RNAs [14, 18–22].

Following the initial observation that EZH2 is over expressed in aggressive myelomas [23, 24], we demonstrated that EZH2 expression is driven by IL-6 and is required for the proliferation of growth-factor-independent human myeloma cell lines (HMCLs) harboring a *ras* mutation [25]. Since publishing these findings, corroborating evidence has accumulated suggesting that EZH2 is aberrantly active in MM and implicating EZH2 as a putative therapeutic target [26–35]. Characterization of recurring EZH2 activating mutations in lymphomas [36] has driven the recent development of several EZH2-specific inhibitors (EZH2i’s: e.g. EPZ6438, GSK126 and UNC1999) which avoid the off-target effects of non-specific histone methyl-transferases inhibitors (i.e. DZNep) previously used to study EZH2 [37–42].

Recent efforts to evaluate the efficacy of EZH2 inhibitors in MM have further described a complex EZH2-mediated regulatory network that modulates the expression of many functionally significant miRNAs, MM-associated oncogenes and cell adhesion pathways [29, 31, 32, 34]. Despite these findings, specific mechanisms of EZH2i-mediated cytotoxicity in HMCLs and biomarkers that distinguish EZH2i-sensitive myelomas remain elusive. Further, it is not clear that EZH2 inhibition is an effective treatment strategy in all myelomas.

In the present study, we profile a large panel of HMCLs for EZH2i efficacy. We found that only a subset of HMCLs respond to single agent EZH2i, but all HMCLs respond to combination treatment with added HDAC inhibition. Additionally, comprehensive transcriptomic profiling of combination treatment reveals substantial changes in oncogenic pathways.

## RESULTS

### EZH2 inhibition reduces viability in a subset of human myeloma cell lines

**Figure 1:**
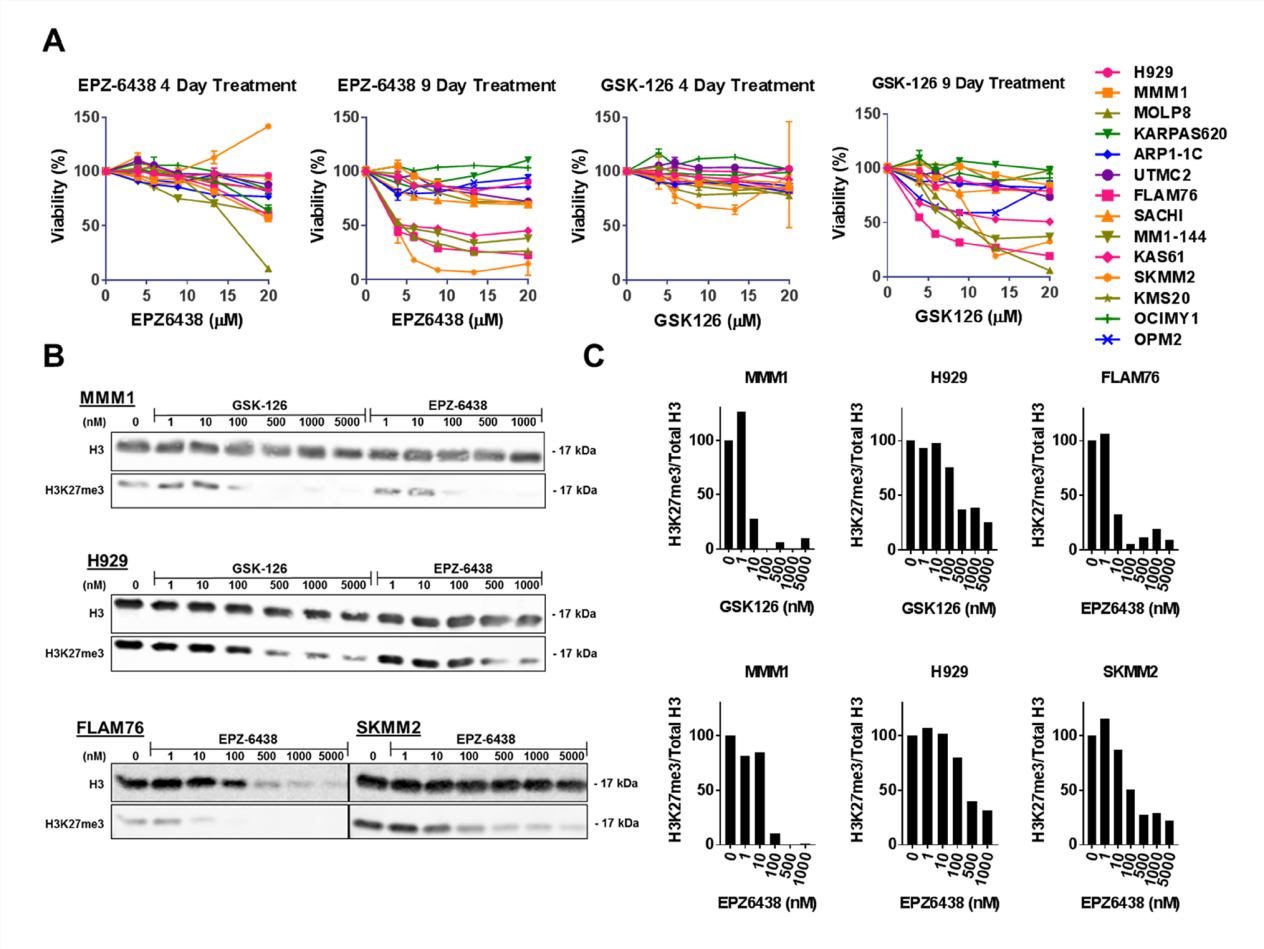
EZH2 inhibition induces H3K27 demethylation in all HMCLs and decreases viability in a subset of HMCLs (**A**) A panel of 14 HMCLs were treated with a concentration range of EZH2 inhibitors EPZ-6438 and GSK-126 for either 4 or 9 days. Viability was measured with CellTiter-Glo^®^ (Promega) assays and normalized to untreated controls. (**B**) H3K27 demethylation was quantified after a 6 day treatment with a range of EZH2 inhibitors in two EZH2i-sensitive (FLAM76 and SKMM2) and two EZH2i-resistant (MMM1 and H929) HMCLs. H3K27me3 was quantified by western blot where total histone 3 (mouse anti-H3; CST#3638) and H3K27me3 (rabbit anti-H3K27me3; CST#9733) were simultaneously quantified via a LI-COR^®^ fluorescence reader. Relative densitometry (**C**) was calculated for each EZH2i concentration and normalized to the untreated control. All error bars represent SEM between biological replicates.

To evaluate the single agent efficacy of EZH2 inhibition as an anti-MM therapeutic strategy we treated a panel of 14 human myeloma cell lines (HMCLs) with the selective EZH2 inhibitors (EZH2i’s) EPZ-6438 and GSK-126. Treatment with these compounds for 4 days or less was insufficient to induce substantial reduction in viability measured via CellTiter-Glo^®^. After 9 days of treatment, both compounds produced a consistent single agent response in a subset of cell lines (Figure 1A). These EZH2i sensitive cell lines demonstrated sensitivity at doses in the low micromolar range within a timeline consistent with others’ observations [39]. We also tested the EZH1/2 dual inhibitor UNC1999 [42] in many of these HMCLs and observed very similar cytotoxic responses compared with EPZ-6438 and GSK-126 and no added sensitivity in EZH2i resistant cell lines upon dual inhibition (data not shown).

To determine whether the lack of response in some HMCLs was due to a lack of target inhibition we extracted histones from treated cell lines to measure the relative abundance of global tri-methylated H3K27; a histone modification sufficient to measure global EZH2 catalytic activity [43]. Western blotting was performed on histones extracted from HMCLs treated with EZH2i’s for 6 days. Dual fluorescent labelling of total H3 and H3K27me3 (Figure 1B) allowed us to quantify (Figure 1C) the relative change in H3K27 de-methylation at different doses relative to an untreated control. Both EZH2i-resistant HMCLs (MMM1 and H929) and EZH2i–sensitive HMCLs (FLAM76 and SKMM2) showed a large decrease in the relative abundance of H3K27me3 at doses well below 1 μM, regardless of the effect on viability. Flam76 is a particularly sensitive cell line that is among the fastest to demonstrate viability loss after EZH2i treatment. This loss in viability explains the apparent decrease in detection of total H3 at higher EPZ-6438 doses. It is interesting to note that this loss of H3 detection occurs at higher doses (100–500 nM) than doses required to reduce relative H3K27me3 (10–100 nM).

### EZH2 inhibitor pre-treatment synergistically enhances sensitivity to the pan-HDAC inhibitor panobinostat

**Figure 2:**
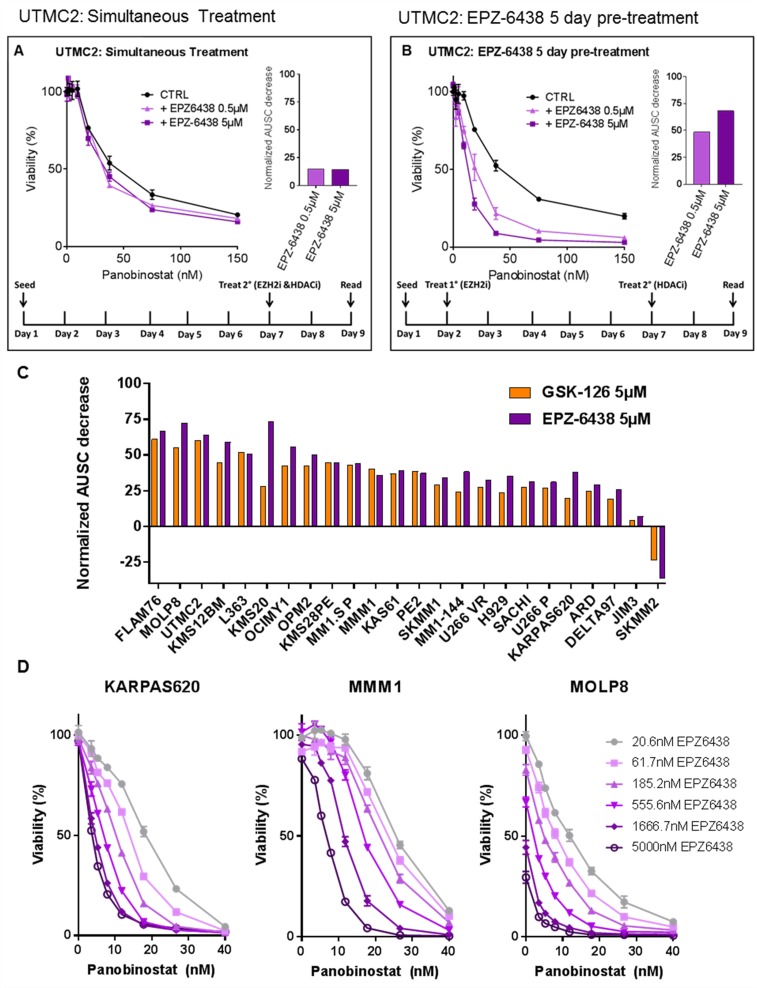
EZH2 inhibitor pre-treatment sensitizes HMCLs to panobinostat in a dose-dependent manner HMCLs were treated with a combination of the pan-HDAC inhibitor panobinostat and EZH2 inhibitors GSK-126 or EPZ-6438. Viability was measured via CellTiter-Glo^®^ and normalized to untreated controls. Two treatment schedules (represented by schematics in (**A**) and (**B**)) were applied and data is represented in the HMCL UTMC2 where EPZ-6438 was either combined with panobinostat simultaneously (A) or 5 days prior to panobinostat (B). Bar plots represent a measurement of synergy quantified by the decrease in the area under the survival curve (AUSC) between panobinostat single agent treatment and combination treatment (where AUSC of each EZH2i+pan/CTRL+pan dose response curve is normalized separately to isolate the shape of the curve from single agent EZH2i toxicity). (**C**) A panel of HMCLs (*n* = 24) were treated with panobinostat for 48hrs after a 4-day pre-treatment with either GSK-126 or EPZ-6438. The resulting synergy is represented as decrease in normalized AUSC across the HMCL panel. (**D**) Three HMCLs representing three levels of EZH2i sensitivity (none, minimal and strong) were pre-treated with a range of EPZ-6438 concentrations for 7 days followed by treatment with a constant range of panobinostat for 48hrs. Viability was measured via CellTiter-Glo^®^. All error bars represent SEM between biological replicates.

Despite heterogeneous HMCL response to EZH2i’s, consistent changes in global H3K27 methylation led us to consider that global epigenetic changes induced by EZH2i’s may sensitize HMCLs to other anti-MM compounds regardless of EZH2i single-agent response. To test this, we treated HMCLs with EZH2i’s in combination with several classes of compounds including proteasome inhibitors, immunomodulatory compounds and glucocorticoid receptor agonists, all of which failed to demonstrate consistent synergistic toxicity with EZH2i’s (data not shown). The pan-HDAC inhibitor panobinostat (Novartis), however, did consistently demonstrate a synergistic effect on HMCL viability. Initially, we found that simultaneously treating HMCLs with panobinostat and EZH2 inhibitors had little synergistic effect (Figure 2A). Pre-treating HMCLs with EZH2i’s for several days, however, strongly enhanced the cytotoxicity of panobinostat (Figure 2B). This was evident even in cases where the single agent EZH2i had no significant effect on viability. We further confirmed that relative loss in viability was cytotoxicity by measuring viability after combination treatment using CellTiter-Glo^®^ in tandem with propidium iodine exclusion staining and flow cytometry (Supplementary Figure 1).

Many HMCLs lack a single agent response to EZH2i’s and therefore we were unable to quantitatively compare this synergistic interaction across a panel of HMCLs using the common Chou-Talalay method for generating combination index plots [44]. We chose instead to compare synergy by calculating the relative drop in the area under the survival curve (AUSC) between dose response curves of panobinostat alone and panobinostat combined with a fixed dose of EZH2i (each curve normalized to untreated or EZH2i-only controls) (Figure 2A, 2B). We systematically evaluated the normalized panobinostat AUSC decrease produced by pre-treatment with either EPZ-6438 or GSK-126 across a panel of 24 HMCLs (Figure 2C). We found that pre-treatment with EZH2i’s strongly enhance the toxicity of panobinostat in almost all cases with consistent results between the two EZH2i’s. We additionally compared the effects of simultaneous EPZ-6438 treatment vs EPZ-6438 pre-treatment on panobinostat toxicity in the same HMCL panel (Supplementary Figure 2). Pre-treatment with EZH2i’s was nearly always more effective. One drawback to the AUSC decrease metric is that in a few cases where the EZH2i single agent response is particularly strong, normalization can exaggerate the change in the shape of the curve and therefore exaggerate the AUSC change or suggest antagonism (i.e. SKMM2 and FLAM76). Despite this, it was clear that pre-treatment with EZH2i’s had a strong dose-dependent effect (Figure 2D) on panobinostat efficacy regardless of EZH2i single agent toxicity across nearly all HMCLs tested.

Having evaluated EZH2i sensitivity in several panel experiments we identified the following HMCLs as having demonstrated consistent EZH2i single agent sensitivity: SKMM2, FLAM76, KMS12BM, L363, MOLP8, MM1-144, KAS61, MM1.S P and MM1.S VR. Overall our data did not suggest any trends between EZH2i sensitive and resistant HMCLs based on characterized genomic lesions including t(4;14), RAS mutation status and UTX/KDM6A mutation status (Supplementary Table 1).

### EPZ-6438 induces robust transcriptomic change as a single agent and in combination with panobinostat

EZH2 and HDACs are both epigenetic regulators known to affect the expression of thousands of genes. We sought to determine if the enhanced cytotoxic response of the EZH2i/panobinostat combination is due to enhanced changes in the expression of a shared set of genes or if the combination produced a large set of gene expression changes that are unique to the combination. We selected 6 HMCLs to sample and screen for the ideal conditions to quantify transcriptomic changes via RNA-sequencing (RNA-seq): MMM1, SACHI, SKMM2, KMS20, KARPAS620 and FLAM76. These HMCLs were selected to represent EZH2i-sensitivity (SKMM2 & FLAM76) and EZH2i-resistance (MMM1, KARPAS620, KMS20 and SACHI). Samples were collected during days 0, 1, 2, 3, 4, 5.5 and 7 during treatment where EPZ-6438 (500 nM or 5 μM) or media was added at day 0 and panobinostat (two sub-IC50 concentrations per line experimentally determined during EZH2i pre-treatment) was added at day 4 as a single agent or as a combination with EPZ-6438 pre-treatment. We systematically evaluated these samples for viability and relative H3K27me3 levels (Supplementary Figure 3) to identify the optimal doses and time points to submit paired samples for RNA-seq. Our results showed that demethylation of H3K27 was complete within the first three days regardless of EPZ-6438 dose. At that time SKMM2 and FLAM76 also began to show a cytotoxic response to EPZ-6438. We chose to submit replicates for sequencing from MMM1 and FLAM76. We chose days 1, 4 and 5.5 that were treated with 5 μM for EPZ-6438 and 3 nM/20 nM panobinostat (FLAM76/MMM1 respectively).

**Figure 3:**
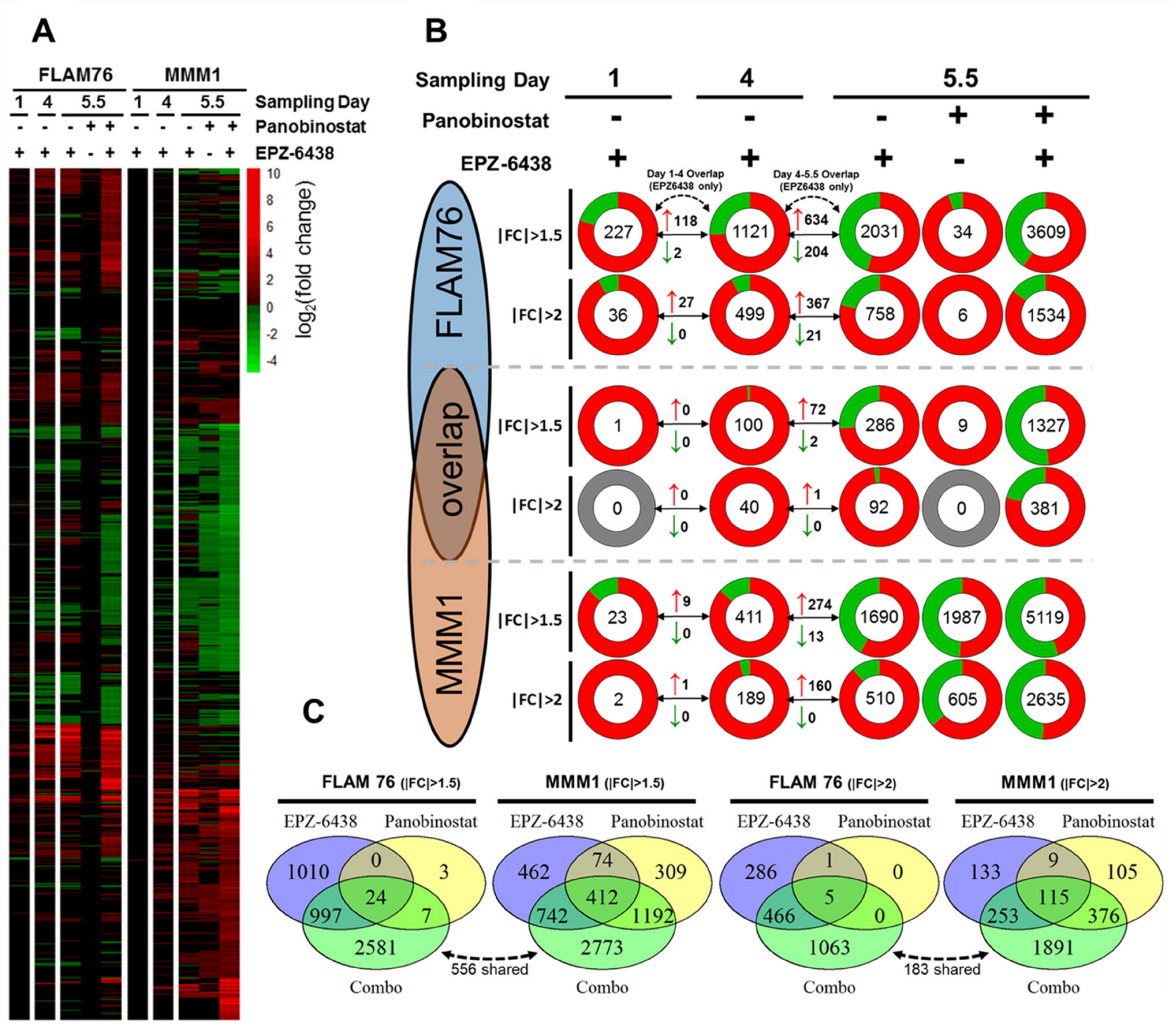
Transcriptomic profiling of EPZ-6438/panobinostat single agents and combination (**A**) All significant (FDR < 0.05, FPKM ≥ 1) gene expression changes for two HMCLs (FLAM76 & MMM1) treated with EPZ-6438 (5 μM for 1, 4 or 5.5 days) and/or panobinostat (FLAM76-3 nM;MMM1-20 nM for 1.5 days after 4 days EPZ-6438/media pre-treatment). Infinite/negative-infinite fold change values (i.e. 0 FPKM relative to 10 FPKM) display the same color saturation as the finite minimum or maximum fold change value. (**B**) Pie charts each representing the total number (center) of upregulated (red) and downregulated (green) genes (FDR < 0.05, FPKM ≥ 1) for each treatment condition. At each condition two fold change thresholds are displayed (|FC|≥2 above and |FC|≥2 below) for both HMCLs as well as for the overlap in significant gene expression changes for each condition/threshold between the two HMCLs. Arrowed lines between sampling days display the number of upregulated (red arrow) and downregulated (green arrow) genes shared between the three different EPZ-6438 single agent sampling times. (**C**) Venn Diagrams displaying genes shared between the day 5.5 EPZ-6438, panobinostat and combo differential expression conditions as well as the genes unique to the combination. The dotted arrow/number represent genes unique to the combination that are shared between the two HMCLs.

RNA-seq revealed large transcriptomic changes induced by EPZ-6438 (Figure 3A, 3B). Full differential expression data for each condition is provided as a supplemental file (Supplementary File 1). Transcriptomic changes were minimal after one day of EZH2i treatment. This was expected given the time required for EZH2i-induced histone demethylation. 4 Days of treatment with EPZ-6438 produced much more substantial gene expression changes with a clear bias towards global upregulation of gene expression in both HMCLs, which was expected following inhibition of a negative regulator of transcription. Gene expression changes that appeared on day 5.5 of treatment were roughly between two and three times the number of differentially expressed genes seen at Day 4. The magnitude of this continued change was surprising, as we did not expect additional deregulation to occur days after the global H3K27me3 levels had reached a minimum. To confirm the specificity of our sequencing between different time points we compared the number of genes conserved between different EPZ-6438 single agent treatment times and found that most of the genes identified in an earlier time point also appeared at the following time point (Figure 3B).

Unfortunately, at the doses used, panobinostat only induced substantial gene expression changes in MMM1. While FLAM76 demonstrated little to no transcriptomic perturbation from panobinostat alone, the combination roughly doubled the number of differentially expressed genes measured from EPZ-6438 alone (758 to 1534 genes with at least a 2-fold expression change). MMM1 additionally demonstrated a very large increase in the number of genes effected by the combination over the two single agents. Comparing the overlap between the two single agent conditions with the combination it is clear that a large portion of differentially expressed genes identified in the combination are unique to the combination (Figure 3C). In both cell lines, at a fold change threshold of ±2 roughly 2/3 of differentially expressed genes in the combination condition were unique to the combination.

We were surprised at the low degree of overlap (consistently below 20–25%) between the two cell lines at all conditions examined. The 758 (FLAM76) and 510 (MMM1) differentially expressed genes at day 5.5 of EPZ-6438 single agent treatment only had 92 overlapping genes. Additionally, there were only 183 genes shared between the 1891(FLAM76) and 1063(MMM1) genes unique to the combination. Another recent study using microarray transcriptomic analysis profiling different MM cell lines in response to EZH2 inhibition also noted little consistency in the magnitude and content of transcriptomic response [29]. It may be that the substantial epigenetic heterogeneity between patient tumors may not yield a predictable gene expression profile in response to EZH2 inhibition.

### Network analysis of EZH2i/HDACi transcriptomic profiles reveals highly enriched cancer-related pathways and regulators

**Figure 4:**
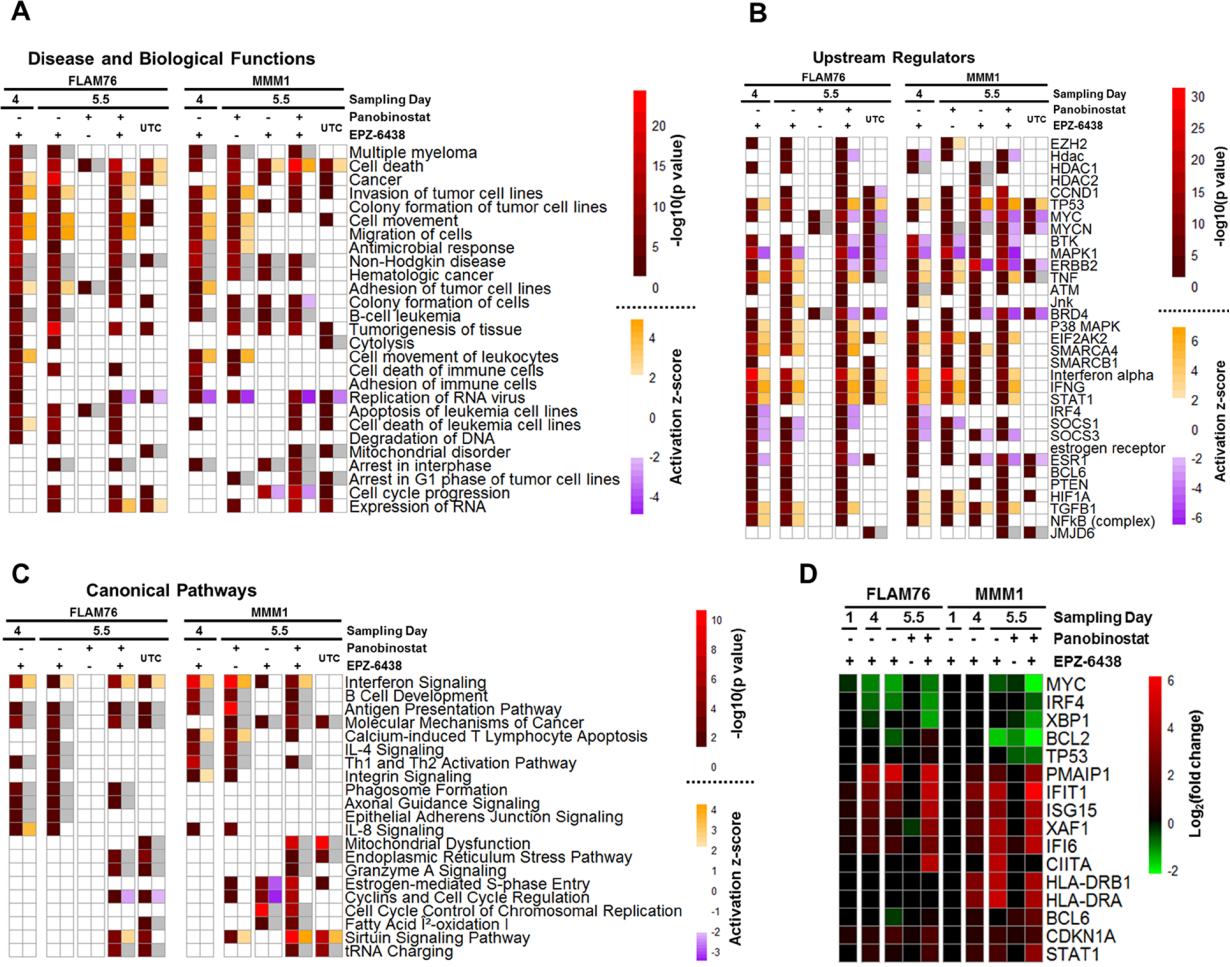
Ingenuity pathway analysis of EPZ-6438-, panobinostat-, and combination-induced gene expression changes Filtered differential gene expression profiles (FPKM≥1, |FC|≥2,FDR<0.05) for two HMCLs (FLAM76 & MMM1) treated with EPZ-6438 (μM), panobinostat (FLAM76-3 nM;MMM1-20 nM) or the combination (relative to time-matched untreated control) were subjected to Ingenuity Pathway Analysis (IPA). Heat maps represent selected top results from different types of IPA analysis: (**A**) predicted upstream regulators (genes, groups or complexes), (**B**) enriched disease and biological functions and (**C**) enriched canonical pathways. Each differential expression condition is represented by two columns. The left column displays the –log10(*p*-value) returned by IPA for enrichment and the right column displays the predicted activation z-score (when applicable; not all predictions have activation directionality). Analysis of unique-to-combination (UTC) genes subsetted from the combination condition for each line is also displayed. White heat map cells represent a lack of significant enrichment/prediction (*p* > 0.05 and |z|<2 respectively). Grey heat map cells represent missing activation z-scores when *p*-values are significant. (**D**) differential expression (RNA-seq: log2 fold change) across all conditions for selected genes pertinent to top IPA hits and discussed in text. Only gene expression changes significant at FDR < 0.05 and |fc|>1.5 are displayed.

The magnitude of transcriptomic change in HMCLs treated with EPZ-6438, panobinostat and the combination required network analysis to identify higher order changes in established molecular pathways and functions. Ingenuity Pathway Analysis (IPA) [45] is a network analysis platform that can be queried with gene expression changes and return enriched network information from the QIAGEN knowledge base. Specifically, we considered significant enrichment of predicted upstream regulators (genes, groups or complexes), diseases/biological functions, and canonical pathways. We submitted filtered gene expression profiles (FDR < 0.05 and |FC|>2) for all differential expression measurements and have included catalogs of all significant (*p* < 0.05) hits returned (Supplementary Files 2–4). We compiled many of the top, contextually relevant hits into heat maps for each of the three analysis categories mentioned above (Figure 4A–4C).

It has been proposed that EZH2 promotes MM development by regulating the expression of numerous oncogenes and tumor suppressors [29–31, 33]. Consistent with this, we observed that many of the upstream regulators predicted from our transcriptomic profiling are key in promoting MM development. For example, CCND1, a core regulator of cell cycle progression, has recurrent mutations in MM and is a highly enriched as a predicted upstream regulator in our transcriptomic profiles. MYC, an aberrantly expressed transcription factor in many cancers including MM [46], is enriched in most of our transcriptomic profiles, has significantly lowered expression (Figure 4D) upon treatment with single agent EPZ-6438/combination in both HMCLs, and is strongly predicted to be deactivated upon combination treatment in both lines. Several additional hits have also been previously proposed to directly or indirectly interact with EZH2 including NFKB [47], STAT1 [48], MYC [49, 50], TP53 [15, 47] and SMARCA4 [51]. IRF4, a late B-cell transcription factor, has recently been shown to facilitate EZH2i-sensitivity through BCL6-mediated downregulation in HMCLs harboring a UTX/KDM6A mutant background [34]. BCL6 is consistently enriched as a predicted upstream regulator in our data and IRF4 expression is downregulated upon combination treatment in both cell lines. It may be possible that the enhanced cytotoxicity of the combination treatment is due, at least in part, to synergistic regulation of key transcription factors such as IRF4 and MYC. Neither FLAM76 nor MMM1 have any known UTX/KDM6A mutations (J. Keats, personal communication) (Supplementary Table 1).

Several upstream regulator hits had a strong prediction of activation/deactivation. For example TP53, TNF, IFNA, IFNG, STAT1 and EIF2AK2 were all predicted as being strongly activated in most conditions suggesting a decreased oncogenic state and increased sensitivity to pro-apoptotic signaling. Examples of regulators with predicted deactivation included BTK, MAPK1 and IRF4. BTK, a kinase critical for B-cell development, is a putative target in several cancers and BTK inhibitors have been shown to act synergistically with HDAC inhibitors in pre-clinical models of lymphoma [52]. While the modulation of these regulators may not be enough to drive cytotoxicity alone, they suggest a general reduction of the pro-growth/anti-apoptotic state of malignant plasma cells.

The diseases and biological functions output from IPA highlighted gene ontology enrichment in many expected areas including hematologic cancers and cell death. Many key genes involved in cell death had some of the highest fold-change expression differences and suggested an increased pro-apoptotic state in single agent EPZ-6438 and to an even higher degree in the combination. These genes included the upregulation of PMAIP1 (NOXA), XAF1 and CDKN1A (p21) and the downregulation of BCL2 and XBP1 (Figure 4D). Terms related to cell adhesion and movement were highly represented. Modulation of cell adhesion has previously been shown to be a consequence of EZH2 inhibition in MM [29].

Canonical pathway analysis yielded many results that reflected some of the same genes enriched in upstream regulators. Some of the strongest and most consistent enrichments included the interferon signaling pathway and the antigen presentation pathway, both of which were enriched after EPZ-6438 single agent treatment and the combination and have been shown to be directly modulated by EZH2 [48]. The interferon pathway has long been considered a target for therapeutic activation in MM [53]. Enrichment of this pathway was centered on the upregulation of STAT1 and most of its downstream promotor targets. The antigen presentation pathway showed a consistent increase in the upstream transcriptional coactivator CIITA and downstream MHC class II genes (Figure 4D). MHCII genes were among others enriched in the B-cell development pathway where other B-cell markers were upregulated.

While there were many consistent enrichments observed with EPZ-6438 single-agent treatment between lines there were few similarities when genes unique to the combination were submitted to IPA. A few exceptions to this include the predicted upregulation of the ‘sirtuin signaling pathway’ and enrichment of the ‘Endoplasmic Reticulum Stress Pathway’, ‘Mitochondrial Dysfunction’ and ‘tRNA Charging’ pathways. Another consistency between both unique-to-combination genes was the strong predicted activation of cell death and apoptosis related ontology terms.

In general, network analysis of EZH2i-induced gene expression changes revealed a consistent modulation of cancer-related pathways in a manner suggesting a less growth-promoting state. The combination with panobinostat indicated strong predictions of cell-stress/death in addition to further perturbation of the pathways identified in the single agents. While many of these factors were previously known to be downstream of EZH2 inhibition, the combination with panobinostat illuminated an enhanced augmentation of tumor-promoting pathways as well as several enriched results unique to the combination.

## DISCUSSION

Recent development of EZH2-specific inhibitors has prompted several studies evaluating the efficacy of EZH2 inhibition in HMCLs. Several of these studies have identified significant pathways and regulators that are modulated in HMCLs upon EZH2 inhibition including contextually relevant oncogenes/tumor suppressors [29, 31, 32, 34], novel miRNAs [31], cell-to-cell adhesion/mobility [29] and dysregulation of cell cycle control [33]. Our evaluation of a large panel of HMCLs for single agent cytotoxic response to EPZ-6438 and GSK-126 recapitulated previously described dose and temporal thresholds for cytotoxicity in HMCLs. Other studies that have evaluated EZH2i’s in HMCLs have measured baseline EZH2 protein levels and did not demonstrate a correlation with single agent sensitivity [29, 33]. Some recent studies have proposed that certain subsets of MM are sensitive to EZH2 inhibition such as MM cases harboring recurring UTX/KDM6A loss-of-function mutations or recurring t(4:14) translocation, both of which are known to directly impact modification EZH2’s target residue and alter EZH2 distribution respectively [34]. The relative sensitivity and resistance for HMCLs that were shared between our evaluation and that of others was generally consistent. In HMCLs not examined by others, predictions of sensitivity based on subtype were consistent in some cases (sensitivity in UTX mutant KMS12, L363 and t(4:14) containing KAS61) and inconsistent in others (resistance in t(4:14) containing OPM2, PE2, H929, JIM3 as well as UTMC2 that contains both t(4:14) and UTX lesions) (Supplementary Table 1). As speculated by others [34], these specific lesion may be sufficient to distinguish sensitivity in certain genomic/epigenomic contexts, however other factors clearly play a role in effecting single agent sensitivity.

To our knowledge, we are the first to report enhancement of HMCL sensitivity to panobinostat via EZH2i pre-treatment. Panobinostat has recently been approved for use in refractory MM, however its therapeutic benefit has been modest [9]. Therefore, combination therapies that enhance HDACi efficacy could have great therapeutic benefit. We found that this synergistic interaction did not require EZH2i single agent sensitivity. This synergistic interaction has been explored in other cancer contexts [54–56] however the combination has yet to be applied in any clinical trials. Encouragingly, one study found that combining panobinostat with a non-specific inhibitor of methyltransferase activity (DZNep) was tolerated in a murine xenograft model of AML [57] suggesting that EZH2i/panobinostat combination may not produce undue *in vivo* toxicity.

We were surprised by how many EPZ-6438-induced transcriptomic changes were observed well after global H3K27 levels had reached a minimum. Extensive global/temporal profiling of chromatin will be required to determine if these final expression changes are direct effects of EZH2i or if they are rather pleiotropic fallout from the substantial epigenetic modification. We have postulated that upregulating the expression of so many genes may result in some non-specific toxicity. This could, in part, explain consistent toxicity of the combination treatment despite an apparent lack of overlap between differentially expressed genes. With a more lenient 1.5 fold-change threshold, combination treatment in FLAM76 and MMM1 showed as much as a roughly 15% and 22% significant differential expression of the queried genome. While the contribution of non-specific transcriptomic stress remains speculative, it is clear is that the combination of the two inhibitors upregulated a large set of genes that were unique to the combination. This suggests that PRC2 and HDACs likely cooperate to silence a large portion of the genome and that this cooperation may be essential for the survival of myelomas that exploit aberrant PRC2 activity.

Our network analysis largely corroborated findings that EZH2 inhibition leads to a robust upregulation of tumor suppressors and concomitant downregulation of oncogenic pathways. These pathways included key regulators of cell-to-cell interaction, antigen presentation, differentiation, apoptosis, cell cycle progression, metabolism and central signaling nodes such as MYC and TP53. MYC, a classic anti-cancer target for which there is no selective small compound inhibitor, seems to be particularly implicated in recent literature describing the anti-cancer effects of EZH2 inhibition [15, 31, 34, 49, 50]. It remains unclear if these factors and pathways directly induce cytotoxicity in combination treatment or if the combined transcriptomic change pushes HMCLs towards a more apoptosis-permissive state that is perturbed by the direct toxicity of panobinostat.

The magnitude of transcriptomic change and network analysis hits, both shared and distinct between the two lines, in addition to the low degree of overlap between the two lines presents a challenge in discerning definitive biomarkers for EZH2i sensitivity. Any attempt to define a consistent consensus EZH2i gene expression profile in HMCLs or to identify biomarkers for sensitivity would require a much more exhaustive transcriptomic profiling of a large panel of HMCLs. Even in that case, we speculate, as others have [29], that identifying a predictive signature or single sensitivity biomarker for a highly networked regulator targeting thousands of genes in an extremely epigenetically heterogeneous disease background is a dubious prospect. Despite these challenges towards defining the scope of PRC2/HDAC interaction specific to MM, accumulating evidence suggests a generalized effect including downregulation of oncogenic pathways and upregulation of tumor suppressors. This leads to either direct cytotoxicity or sensitization of HMCLs to combination therapies in a targeted manner.

In conclusion, our data suggests that while only a subset of human myeloma cell lines respond to EZH2 inhibition, nearly all lines tested were effectively targeted for cell death through a synergistic combination of panobinostat and EZH2 inhibitor pre-treatment. This combination was effective at lowering the therapeutic threshold of panobinostat even in cases where there was no single agent EZH2 inhibitor response. Transcriptomic analysis of single agents and combination treatments corroborates the regulation of many oncogenic pathways towards a less growth-promoting state and reveals a large transcriptomic response unique to the drug combination. These data support the further evaluation of therapeutic combination to broadly target aggressive MM in *in vivo* and clinical contexts.

## MATERIALS AND METHODS

### Drugs

Panobinostat/LBH-589 (Novartis; Basel, Switzerland), GSK-126 (GlaxoSmithKline; Brentford, U.K.) and Tazemetostat/EPZ-6438 (Epizyme; Cambridge, MA) were purchased from Selleckchem (panobinostat and EPZ-6438) and Cayman Chemical (GSK-126). All drugs were dissolved in DMSO (Sigma-Aldrich; St. Louis, MO) and stored at −20 ° C.

### Cell culture and viability assays

Cell culture conditions for HMCLs used are previously described [58]. HMCLs were seeded at 4 × 10^5^ cells per ml in 96-well plates and were treated with the primary drug (EZH2i) after 24 hrs. Secondary treatment (panobinostat) was added at a small volume (32x) to minimize dilution of the primary treatment. Cell viability was measured using a CellTiter-Glo luminescent viability assay (Promega; Madison, WI) and a Synergy 2 Microplate Reader (BioTek; Winooski, VT). For propidium iodine exclusion assays, plated HMCLs were transferred to round bottom 96-well plates (125 μl/well), pelleted, resuspended in 200 μl PBS containing 2 μg propidium iodine, and propidium iodine staining was quantified using a BD FACSCantoII RUO Flow Cytometer (BD Biosciences; Franklin Lakes, NJ). Area under the survival curve (AUSC) was calculated using the trapezoidal method. Before AUSCs were compared to measure synergy each curve was individually normalized to the zero panobinostat condition. All viability data is normalized to untreated (i.e. media-treated) controls.

### Histone analysis

Histones were isolated using a Histone Extraction Kit (Abcam; Cambridge, U.K.: ab113476). Extracted histone were western blotted for total H3 (CST; Danvers, MA: 96C10) and H3K27Me3 (CST: C36B11). Fluorescently labelled secondary antibodies (LICORE; Lincoln, NE: IRDye^®^ 680RD and 800CW) were quantified with a LI-COR^®^ fluorescence imager and densitometry was quantified in Image J (NIH).

### Transcriptomic profiling and analysis

RNA was extracted (RNeasy Kit; QIAGEN) and stored in RNAlater™ (Invitrogen; Carlsbad, CA) at –80° C. Biological triplicates were subjected to RNA-sequencing (RNA-seq) on a HiSeq 2000 (Illumina; San Diego, CA) using 50bp single-end reads at a depth of >10 million reads per replicate. Sequencing data was subjected to quality control analysis (FastQC), processed (FASTQ and Tophat2(aligned to hg19)), analyzed for differential expression (Cufflinks and Cuffdiff) and visualized (CummeRbund) using the open source, web-based platform Galaxy [59]. Downstream subsetting and analysis was conducted using the R programing language and Ingenuity Pathway Analysis (IPA; QIAGEN; Venlo, Netherlands). Differential expression was considered significant with an FDR < 0.05 and FPKM values ≥ 1 for at least one value (control or treated).

## SUPPLEMENTARY MATERIALS FIGURES AND TABLES










