# Supplementary material for: EZH2 inhibitors sensitize myeloma cell lines to panobinostat resulting in unique combinatorial transcriptomic changes
Source: Oncotarget. 2018 Apr 24;9(31):21930–42. doi: 10.18632/oncotarget.25128 (PMC5955152; doi:10.18632/oncotarget.25128)
Supplement: Supplementary file 1 [file oncotarget-09-21930-s001.pdf]

EZH2 inhibitors sensitize myeloma cell lines to panobinostat resulting in unique combinatorial transcriptomic changes

SUPPLEMENTARY MATERIALS

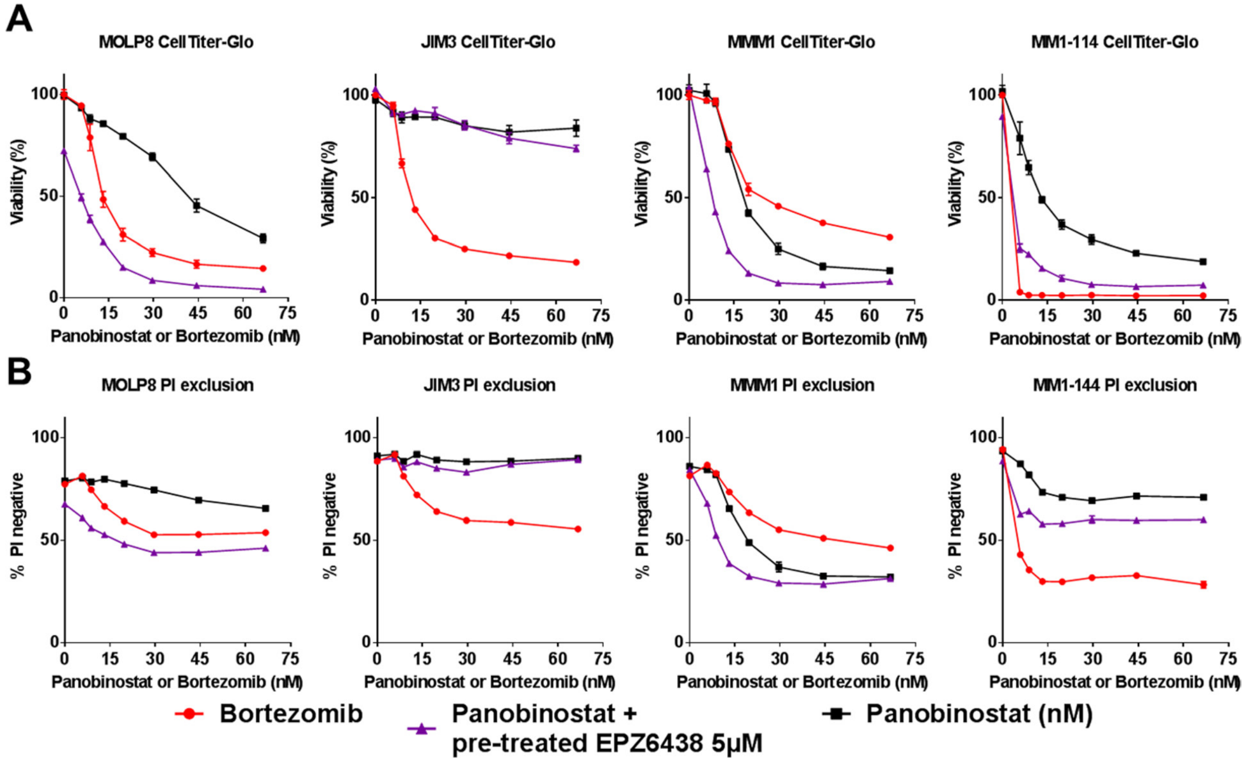

Supplementary Figure 1: Cytotoxicity of EZH2i and panobinostat combination is confirmed via propidium iodine exclusion assay. Four HMCLs seeded in 96-well plates were treated for 48 hrs with either panobinostat or the protesome inhibitor bortezomib (Takeda). These treatments were preceded by a 5-day treatment with either 5 µM EPZ-6438 (panobinostat only) or media. After the 48 hr secondary treatment plates were sampled and assayed for viability by both CellTiter-Glo® (**A**) and propidium iodine (PI) staining followed by flow cytometry (**B**) in tandem. All error bars represent SEM between biological replicates.

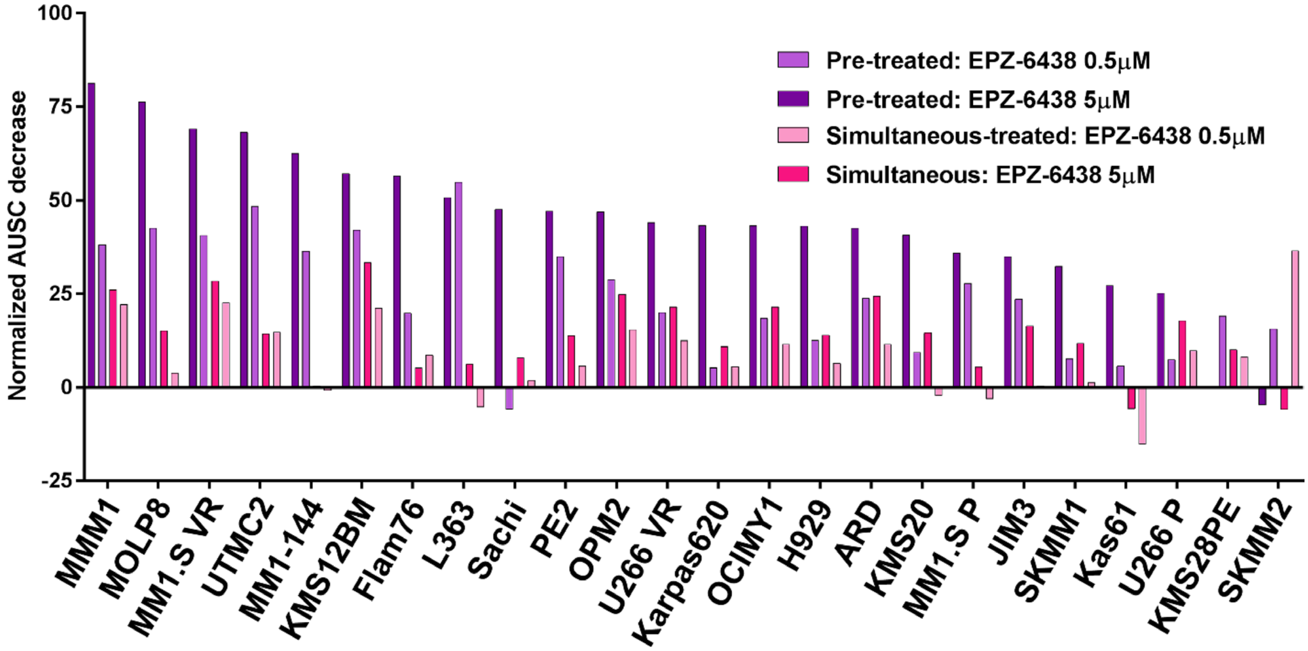

Supplementary Figure 2: EPZ-6438 sensitizing of HMCLs to panobinostat is consistently more effective as a pre-treatment across an HMCL panel. 24 HMCLs were either pre-treated with EPZ-6438 for 5 days followed by a 48hr panobinostat treatment ("pre-treated") or they were pre-treated with media for 5 days followed by a 48hr simultaneous treatment with EPZ-6438 and panobinostat. In both cases two doses of EPZ-6438 were evaluated (0.5μM & 5μM). Viability was measured via CellTiter-Glo® and synergy is represented by the decrease in normalized AUSC between single agent panobinostat and the combination treatment. Negative values represent an increase in normalized AUSC.

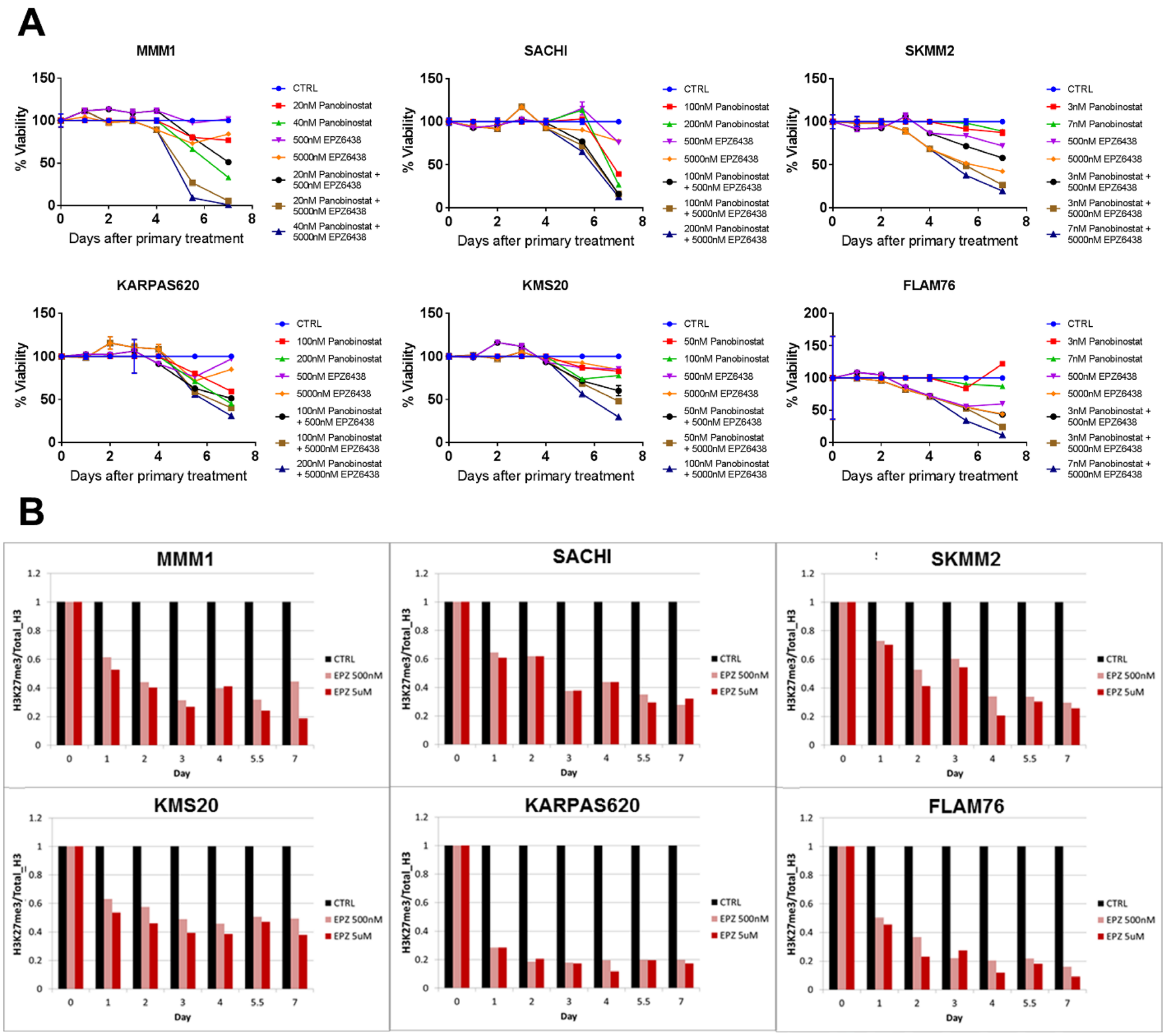

Supplementary Figure 3: Time-course measurement of H3K37 demethylation and cell viability after treatment with EPZ-6438, panobinostat and the combination in 6 HMCLs. 6 HMCLs (MMM1, SACHI, SKMM2, KMS20, KARPAS620 and FLAM76) were sampled at 0, 1, 2, 3, 4, 5.5 and 7 days post-treatment with 500nM EPZ-6438, 5µM EPZ-6438 or media (untreated control). Cells additionally treated with panobinostat (single agent and combined with EPZ-6438 4 day pre-treatment) on day 4 in this sampling schedule were harvested on days 5.5 and 7. Samples were either (**A**) immediately measured for viability via CellTiter-Glo® (normalized to time-matched untreated control) or subjected to histone-purification and storage at –80° C. Frozen histone preparations were later western blotted and quantified (**B**) for H3K27me3 abundance (each sampling day normalized to total H3).

Supplementary Table 1: List of human myeloma cell lines used in this study with annotated UTX/KDM6A, RAS and t(4;14) status

MM cell line	UTX/KDM6A status	RAS status	t(4;14)
**ARD**	Homozygous mutation	ND	No
**ARP1-1C**	WT	WT	No
**DELTA97**	WT	WT	No
**FLAM76**	WT	WT	No
**H929**	WT	NRAS mutation	Yes
**JIM3**	WT	KRAS mutation	Yes
**KARPAS620**	WT	KRAS mutation	No
**KAS61**	WT	WT	Yes
**KMS12BM**	Mutation	WT	No
**KMS20**	WT	KRAS mutation	No
**KMS28PE**	Homozygous mutation	KRAS heterozygous mutation	Yes
**L363**	Mutation	NRAS mutation	No
**MM1.S**	WT	KRAS mutation	No
**MM1.S VR**	WT	KRAS mutation	No
**MM1-144**	ND	ND	ND
**MMM1**	WT	NRAS mutation	No
**MOLP8**	WT	NRAS mutation	No
**OCIMY1**	WT	KRAS mutation	No
**OPM2**	WT	FGFR3 mutation	Yes
**PE2**	WT	NRAS mutation	Yes
**SACHI**	WT	WT	No
**SKMM1**	WT	NRAS mutation	No
**SKMM2**	WT	WT	No
**U266**	WT	BRAF mutation	No
**U266 VR**	WT	BRAF mutation	No
**UTMC2**	Homozygous mutation	WT	Yes

'ND' = no data. 'WT' = wild type. Zygosity is not specified in every case. HMCL status data is sourced from the Keat's lab repository (http://www.keatslab.org/data-repository) and recently published data [152]. HMCLs demonstrating consistent sensitivity to EZH2 inhibition in our experiments appear above in red.

Supplementary File 1: Full differential expression results from RNA-seq experiments in both cell lines. Each differential expression condition (treatment vs untreated control) is on its own tab. In each tab genes with no detectable expression in either control or treatment have been removed. Infinite (positive or negative) 'log2.fold.change' values are represented by '#NUM!' in .xlsx format. See Supplementary_File_1

Supplementary File 2: Full catalog of Ingenuity Pathway Analysis (QIAGEN) results: canonical pathway analysis. Each differential expression condition (treatment vs untreated control) is on its own tab. Activation z-scores absent when not provided by IPA. See Supplementary_File_2

Supplementary File 3: Full catalog of Ingenuity Pathway Analysis (QIAGEN) results: upstream regulator analysis. Each differential expression condition (treatment vs untreated control) is on its own tab. Activation z -scores absent when not provided by IPA. See Supplementary_File_3

Supplementary File 4: Full catalog of Ingenuity Pathway Analysis (QIAGEN) results: disease and biological functions analysis. Each differential expression condition (treatment vs untreated control) is on its own tab. Activation z -scores absent when not provided by IPA. See Supplementary_File_4
